# Osteoblast-specific inactivation of p53 results in locally increased bone formation

**DOI:** 10.1371/journal.pone.0249894

**Published:** 2021-11-18

**Authors:** Nannan Liao, Till Koehne, Jan Tuckermann, Ioanna Triviai, Michael Amling, Jean-Pierre David, Thorsten Schinke, Julia Luther

**Affiliations:** 1 Department of Osteology and Biomechanics, University Medical Center Hamburg-Eppendorf, Hamburg, Germany; 2 Department of Orthodontics, College of Stomatology, North China University of Science and Technology, Tangshan, China; 3 Department of Orthodontics, University Medical Center Hamburg-Eppendorf, Hamburg, Germany; 4 Institute of Comparative Molecular Endocrinology, University Medical Center Ulm, Ulm, Germany; 5 Department of Stem Cell Transplantation, University Medical Center Hamburg-Eppendorf, Hamburg, Germany; University of Vermont College of Medicine, UNITED STATES

## Abstract

Inactivation of the tumor suppressor p53 (encoded by the *Trp53* gene) is relevant for development and growth of different cancers, including osteosarcoma, a primary bone tumor mostly affecting children and young adolescents. We have previously shown that deficiency of the ribosomal S6 kinase 2 (Rsk2) limits osteosarcoma growth in a transgenic mouse model overexpressing the proto-oncogene *c-Fos*. Our initial aim for the present study was to address the question, if Rsk2 deficiency would also influence osteosarcoma growth in another mouse model. For that purpose, we took advantage of *Trp53*^*fl/fl*^ mice, which were crossed with *Runx2*^*Cre*^ transgenic mice in order to inactivate p53 specifically in osteoblast lineage cells. However, since we unexpectedly identified *Runx2*^*Cre*^-mediated recombination also in the thymus, the majority of 6-month-old *Trp53*^*fl/fl*;*Runx2-Cre*^ (thereafter termed *Trp53*^*Cre*^) animals displayed thymic lymphomas, similar to what has been described for *Trp53*-deficient mice. Since we did not detect osteosarcoma formation at that age, we could not follow our initial aim, but we studied the skeletal phenotype of *Trp53*^*Cre*^ mice, with or without additional Rsk2 deficiency. Here we unexpectedly observed that *Trp53*^*Cre*^ mice display a unique accumulation of trabecular bone in the midshaft region of the femur and the humerus, consistent with its previously established role as a negative regulator of osteoblastogenesis. Since this local bone mass increase in *Trp53*^*Cre*^ mice was significantly reduced by Rsk2 deficiency, we isolated bone marrow cells from the different groups of mice and analyzed their behavior *ex vivo*. Here we observed a remarkable increase of colony formation, osteogenic differentiation and proliferation in *Trp53*^*Cre*^ cultures, which was unaffected by Rsk2 deficiency. Our data thereby confirm a critical and tumorigenesis-independent function of p53 as a key regulator of mesenchymal cell differentiation.

## Introduction

The p53 tumor suppressor is known to regulate the transcription of various genes, which eventually causes specific cellular responses, such as inhibition of proliferation to prevent detrimental effects of genotoxic stress [1]. Mutations of p53 were identified in more than 50% of primary tumors, and heterozygous germline mutations in the p53-encoding gene (*TP53*) are the cause of Li-Fraumeni syndrome, a disease characterized by increased cancer incidence with a 10% risk to develop osteosarcomas [2]. The tumor suppressor function of p53 has also been demonstrated by targeted deletion of the murine p53-encoding gene (*Trp53*), albeit the incidence of specific tumors was slightly different when compared to Li-Fraumeni syndrome [3]. More specifically, the majority of *Trp53*-deficient mice develop lymphomas, which also reduces their lifespan and the development of other cancer types such as osteosarcoma. However, although the tumor spectrum associated with p53 inactivation was remarkably different in *Trp53*-heterozygous mice, our previous skeletal analysis of 12-month-old *Trp53*^*+/-*^ animals revealed that osteosarcoma development was only detectable, when the gene encoding the protein tyrosine phosphatase Rptpζ was deleted simultaneously [4]. Therefore, we took advantage of mice expressing the Cre recombinase under the control of the *Runx2* promoter [5] with the aim to delete p53 selectively in osteoblast progenitor cells to enable osteosarcoma development. The initial purpose of our study was to utilize this conditional p53 inactivation model, to combine it with a deficiency of Rsk2 and to monitor osteosarcoma incidence and growth.

Rsk2 is a broadly expressed serine/threonine kinase, which in response to growth factor stimulation phosphorylates specific proteins, including p53, to influence various cellular functions [6, 7]. Inactivating mutations of human *RSK2*, which is located on the X chromosome, cause Coffin Lowry syndrome, a rare disease characterized by mental and psychomotoric retardation, but also by skeletal abnormalities [8, 9]. The impact of Rsk2 inactivation on the skeleton was also evidenced by the generation and analysis of *Rsk2*^*-/0*^ mice, which display low bone mass due to a cell-autonomous osteoblast defect, but also dental abnormalities, such as alveolar bone loss and impaired cementum formation [10–12]. With respect to osteosarcoma development we have previously shown that Rsk2 deficiency has a remarkable protective influence on tumor growth in *c-Fos*-transgenic mice. More specifically, while *c-Fos*-transgenic mice develop osteosarcomas in different skeletal locations, which strongly increase in size from 1 to 7 months of age, the tumor area did not increase in *Rsk2*-deficient *c-Fos*-transgenic mice [11, 13]. Although these remarkable findings suggested that Rsk2 is essential for osteosarcoma progression, they were not fully unexpected, since c-Fos is one of the known substrates of the Rsk2 kinase activity [14, 15]. Therefore, it was important to address the question if Rsk2 deficiency can also limit osteosarcoma progression in other mouse models.

Here we describe that the majority of 6-month-old *Trp53*^*fl/fl*;*Runx2-Cre*^ (thereafter termed *Trp53*^*Cre*^) mice displayed thymic lymphomas, due to additional *Trp53* recombination in the thymus, whereas osteosarcomas were not detected. Although this unexpected finding did not allow us to analyze the influence of Rsk2 deficiency on osteosarcoma development, we unexpectedly observed a remarkable accumulation of trabecular bone in the femur and humerus midshaft region of *Trp53*^*Cre*^ mice, which did not appear in *Rsk2*-deficient *Trp53*^*Cre*^ mice. Analysis of cultured bone marrow cells from mice of the different genotypes further revealed that *Trp53*^*Cre*^ cultures displayed increased osteogenic differentiation and proliferation, regardless of their *Rsk2* genotype. These data identify p53 as a key regulator of mesenchymal cell differentiation, whose expression in osteoblast progenitors is required to limit physiological bone formation.

## Materials and methods

### Animals

All mice used in this study were male littermates from matings of *Trp53*^*fl/fl*^ (thereafter termed *Trp53*^*fl*^) males and *Trp53*^*Cre*^ females, the latter being heterozygous for the Rsk2-deficient allele (Rps6ka3^tm1.1Kry^ Trp53^tm1Brn^ Tg(Runx2-icre)1Jtuc/Uke). Mice of all genotypes were available and studied previously by our group [5, 11, 16]. Initially, genotyping of *Trp53*^*fl*^ mice was performed with the primers 5’-AGC ACA TAG GAG GCA GAG AC-3’ and 5’-CAC AAA AAC ACG TTA AAC CCA G-3’, amplifying a 370 bp and 288 bp fragment for floxed and wildtype allele of *Trp53*, respectively. Genotyping for *Trp53* recombination at the loxP-sites was performed with primers 5’-CAC AAA AAC ACG TTA AAC CCA G-3’ and 5’-GAA GAC AGA AAA GGG GAG GG-3’, amplifying a 612 bp fragment for the recombined *Trp53* allele. Genotyping for the *Runx2-Cre* transgene was performed using the primers 5’-TGG CTT GCA GGT ACA GGA-3’, 5’-CCA GGA AGA CTG GAA GAA GC-3’ and 5’-GGA GCT GCC GAG TCA ATA AC-3’, which amplify a 780 bp fragment of the endogenous Runx2 gene and a 600 bp fragment of the *Runx2-Cre* transgene, respectively [5]. Genotyping for *Rsk2* was performed using the primers 5’-TTG TTG GTT TAC TTT CTT TCG GTC TG-3’, 5’-AAG ATG ATT GCT TTG CTT AGT TTA-3’, amplifying a 230 bp and 320 bp for the wildtype and mutant Rsk2 allele, respectively [11]. All mice were kept with a 12-hour light/dark cycle and had access to tap water and standard rodent chow (1328P, Altromin Spezialfutter GmbH & Co. KG, Germany) *ad libitum*. Mice were analyzed at a maximum age of 6 months, mostly before the occurrence of any visible symptoms. Mice were anesthetized with an 80% (v/v) CO_2_/O_2_ gas mixture before switching to 100% CO_2_. Sample size was determined by availability, however, with the exception of FACS analysis (S1 Fig), at least 6 mice were analyzed per genotype. Therefore, in this study, 75 mice were used. All animal experiments were approved by the animal facility of the University Medical Center Hamburg-Eppendorf and by the “Behörde für Soziales, Familie, Gesundheit und Verbraucherschutz” (Org869 and 984).

### μCT analysis

For μCT analysis the right femur and the right humerus were dissected from the mice fixed in 3.7% PBS-buffered formaldehyde for 24 h and fixed in the holder (Φ20mm*H75mm). The holder was placed in a μCT 40 desktop cone-beam microCT (Scanco Medical, Swizerland). Anatomical separation of the skull was performed in the same way, but a different holder was used (Φ30mm*H75mm). μCT scanning and analysis were performed with a voxel resolution of 10 μm for bone analysis and 15 μm for dental analysis as previously described using a μCT 40 desktop cone-beam microCT (Scanco Medical, Swizerland) according to standard guidelines [17, 18]. Sample was analyzed in the distal or mid metaphysis in a volume situated 2500 μm to 500 μm proximal of the distal growth plate. A threshold value of 250 was implemented.

### Histology

Skeletons were fixed in 3.7% PBS-buffered formaldehyde for 24 h and subsequently stored in 80% ethanol. For bone histology, the lumbar vertebral bodies L1 to L4 and the right tibia of each mouse were dehydrated in ascending alcohol concentrations and then embedded in methylmetacrylate. Sections of 4 μm thickness were cut in the sagittal plane on a Microtec rotation microtome (Techno-Med GmbH, Germany) and stained by von Kossa/van Gieson, as described previously [17]. Paraffin histology was performed on the skull. The skulls were placed into the 10% EDTA (pH7.4) for at least 4 weeks, followed by embedding in paraffin. Sections of 3.5 μm were cut on a semi-automated rotary microtome (Leica Biosystems Nussloch GmbH) and stained by toluidine blue, as described previously [17]. Histomorphometry was performed according to the ASBMR guidelines [19] using the OsteoMeasure histomorphometry system (Osteometrics Inc., USA).

### Serum analysis

Serum concentrations were measured using commercially available ELISAs for PINP (SEA957Mu, Cloud Clone Corp.), CTX (AC-06F1, Immunodiagnostic Systems), RANKL (MTR00, R&D Systems) and OPG (#MOP00, R&D Systems).

### *Ex vivo* osteogenic differentiation of bone marrow cells

Bone marrow cells were collected by centrifugation from long bones of mice with the four different genotypes. The cells were then plated in 12-well plates at a density of 5x10^6^ cells/well in α-MEM (Sigma-Aldrich, USA) supplemented with 10% (v/v) FBS (American Type Culture Collection, USA) and 100 U/ml penicillin/streptomycin (Life Technologies, USA). To induce osteoblastic differentiation the medium was additionally supplemented with 50 μg/ml ascorbic acid and 10 mM β-glycerophosphate for 10 days. Alizarin red staining and quantification of mineralization was performed as described previously [17].

### Colony formation assay

Bone marrow cells were collected as described above, and 9 x 10^6^ cells were plated into a Ø15 cm dish. Cells were cultured with α-MEM medium (Sigma-Aldrich, USA) supplemented with 15% (v/v) FBS (American Type Culture Collection, USA) and 100 U/ml penicillin/streptomycin (Life Technologies, USA). After 14 days the cultures were washed with PBS and fixed with 3.7% PBS-buffered formaldehyde for 90 seconds. Staining was performed with alkaline phosphatase substrate solution (0.6 mg/ml fast blue RR salt, 0.25% (w/v) naphthol AS-MX phosphate (both Sigma-Aldrich, USA), pH 8.6) for 30 min. All colonies with more than 50 cells were counted. There further, hematoxylin (Sigma-Aldrich, USA) was used to determine the total colony number.

### Proliferation and gene expression

Bone marrow cells were plated in a 96-well plate for proliferation analysis and in 12-well plate for expression analysis. Cells were cultured with α-MEM medium (Sigma-Aldrich, USA) supplemented with 10% (v/v) FBS (American Type Culture Collection, USA) and 100 U/ml penicillin/streptomycin (Life Technologies, USA). For proliferation analysis, the Amersham cell proliferation biotrak Elisa kit (GE Healthcare, UK) was used and measured with a microplate reader at an absorbance of 450 nm within 5 minutes. For gene expression analysis, RNA was isolated using the peqGOLD TriFast^™^ (Darmstadt, DE), according to manufacturer’s instructions. Concentration and quality of RNA were measured using a NanoDrop ND-1000 system (NanoDrop Technology). Complementary DNA synthesis was performed using the Verso cDNA Synthesis Kit (Thermo Fisher). Expression analysis by qRT-PCR was performed using a StepOnePlus system and predesigned TaqMan gene expression assays (Applied Biosystems). *Gapdh* expression was used as an internal control, and presented data are given as relative expression towards *Gapdh*.

### FACS analysis

For fluorescence activated cell sorting (FACS) we isolated bone marrow from long bones of *Trp53*^*fl*^ and *Trp53*^*Cre*^ mice. Cells were stained with fluorescent conjugated antibodies against CD45, CD34, CD11b, CD11c, F4/80, CD3e, CD4 and CD45R (BD Biosciences, San Jose, CA). Analyses were performed using a FACS Aria Fusion (BD Biosciences, San Jose, CA) and data were analyzed using FlowJo software (Tree Star Inc, Ashland, OR).

### Statistical analysis

All data in the manuscript are presented as means ± standard deviations. Data were analyzed using GraphPad Prism Software (Graphpad Software Inc.). Statistical significance for the comparison of four groups was calculated using one-way ANOVA followed by a post hoc comparison with Bonferroni’s method. For the comparison of two groups data were analyzed by two-tailed Student’s t-test using Excel software (Microsoft Corp., Redmond, WA, USA). p-values below 0.05 were considered as statistically significant.

## Results

### *Trp53*^*fl/fl;Runx2-Cre*^ mice develop thymic lymphomas, but no osteosarcomas

**Fig 1:**
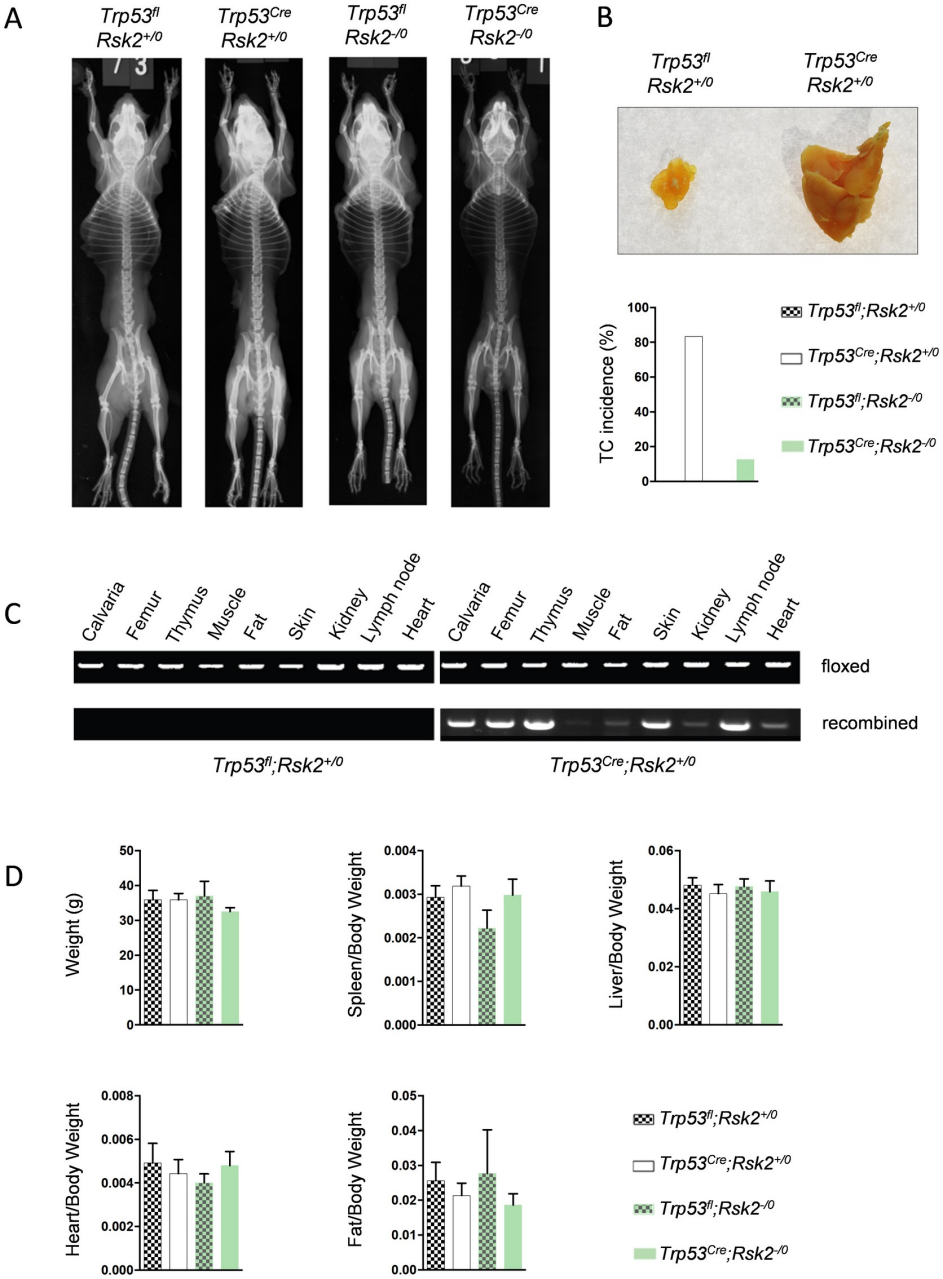
*Trp53*^*Cre*^ mice develop thymic lymphomas, but no osteosarcomas. (A) Representative contact Xrays of 6-month-old male mice with the indicated genotypes. (B) Representative images showing a thymic lymphoma in a 6-month-old *Trp53*^*Cre*^ mouse. Quantification of the thymic lymphoma incidence is given below. (C) Genomic PCR amplification of the floxed and recombined *Trp53* allele with DNA obtained from the indicated tissues of *Trp53*^*fl*^ or *Trp53*^*Cre*^ mice. (D) Quantification of body and organ weights in the different groups of mice. Data represent mean ± SD (n ≥ 6).

For this study we generated and analyzed male littermates with four different genotypes (*Trp53*^*fl;Rsk2+/0*^; *Trp53*^*Cre;Rsk2+/0*^; *Trp53*^*fl*;*Rsk2-/0*^; *Trp53*^*Cre;Rsk2-/0*^). When we analyzed these mice at the age of 6 months we found, by contact Xray, no indication of osteosarcoma development in any genotype (Fig 1A). Unexpectedly however, we identified thymic lymphomas in the majority of *Trp53*^*Cre*^ mice, whereas this incidence was apparently reduced in Rsk2-deficient *Trp53*^*Cre*^ littermates (Fig 1B). We next analyzed the specificity of Cre-mediated recombination in different tissues by genomic PCR. We hereby observed that *Trp53*^*Cre*^ mice displayed recombination within the *Trp53* locus, not only in calvaria and femur (after removal of the bone marrow by centrifugation), but also in skin and thymus (Fig 1C).

Although this finding precluded to study the influence of Rsk2 deficiency on osteosarcoma formation in the *Trp53*^*Cre*^ model, we took of advantage of these animals to analyze a potential impact of p53 inactivation on differentiation of osteoblast lineage cells. In a first step we determined body weight and the weights of different organs (spleen, liver, heart, fat), but we did not detect statistically significant differences between the four groups of mice (Fig 1D). These data suggested that *Trp53*^*Cre*^ mice, despite having thymic lymphomas, do not display a multi-organ pathology, at least not at 6 months of age.

### *Trp53*^*fl/fl;Runx2-Cre*^ mice display increased trabecular bone mass in the femur and humerus diaphysis

**Fig 2:**
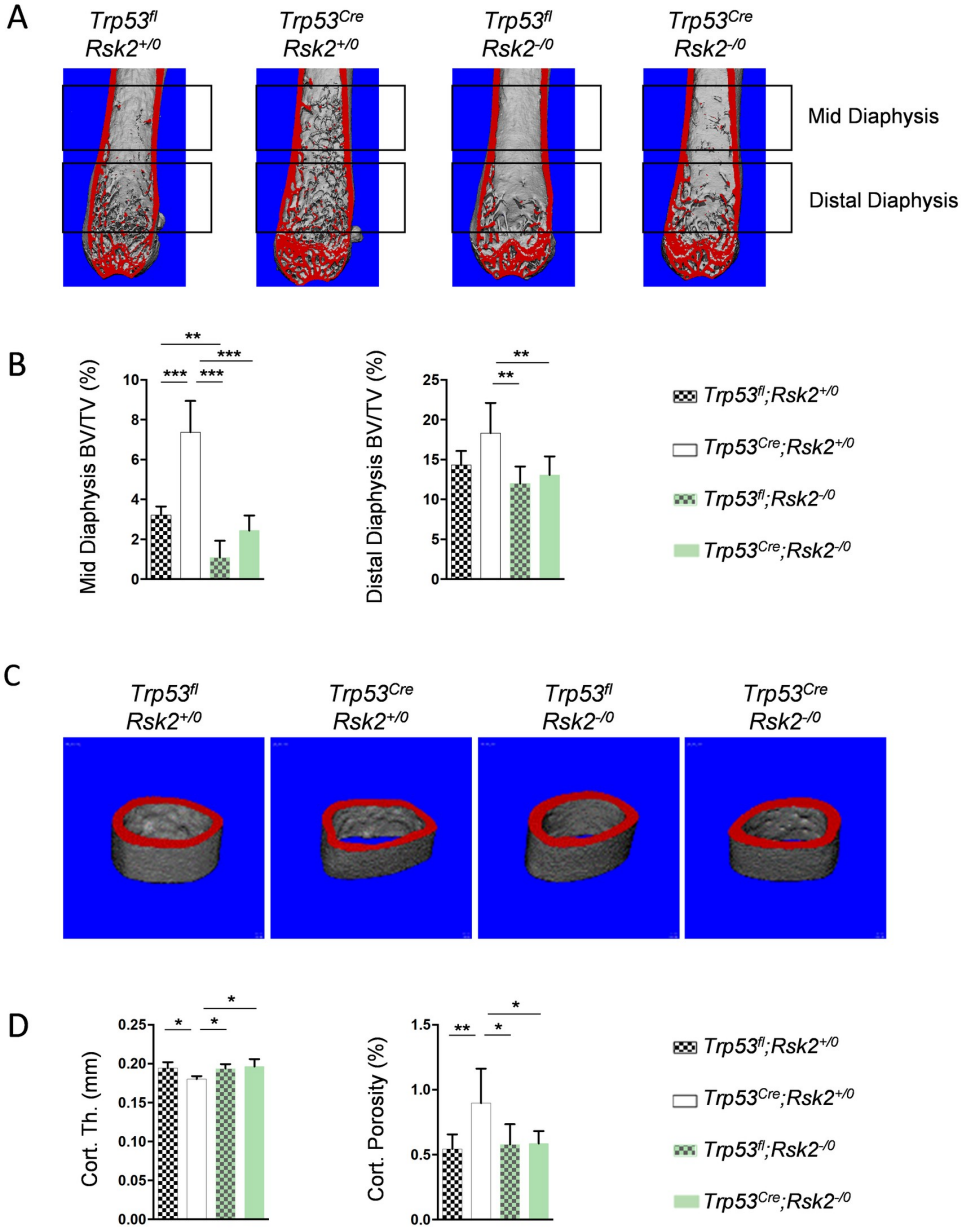
*Trp53*^*Cre*^ mice display increased trabecular bone mass in the femur diaphysis. (A) Representative μCT images of femora from 6-month-old male mice with the indicated genotypes. The highlighted regions were separately analyzed. (B) Quantification of the trabecular bone volume per tissue volume (BV/TV) in the mid diaphysis (left) and the distal diaphysis (right) in 6-month-old male mice with the indicated genotypes. (C) Representative μCT images of the femoral midshaft from the same mice. (D) Quantification of cortical thickness (Cort. Th.) and porosity (Cort. Por.) in the same mice. Data represent mean ± SD (n ≥ 6). Asterisks indicate statistically significant differences (*p<0.05, **p<0.005, ***p<0.0005).

We next analyzed the femur bone architecture in the respective animals by micro computed tomography (μCT) (Fig 2A). Here we identified a remarkable accumulation of trabecular bone in the mid diaphysis specifically in *Trp53*^*Cre*^ mice. Moreover, while the bone volume per tissue volume (BV/TV) was significantly reduced in *Rsk2*-deficient mice, the additional Rsk2 deficiency also corrected the high trabecular bone mass phenotype of *Trp53*^*Cre*^ mice (Fig 2B). There were also subtle changes in the cortical bone compartment observed in *Trp53*^*Cre*^ mice (Fig 2C). More specifically, these animals displayed moderately reduced cortical thickness and increased cortical porosity, and again this was not observed in *Trp53*^*Cre;Rsk2-/0*^ littermates (Fig 2D).

**Fig 3:**
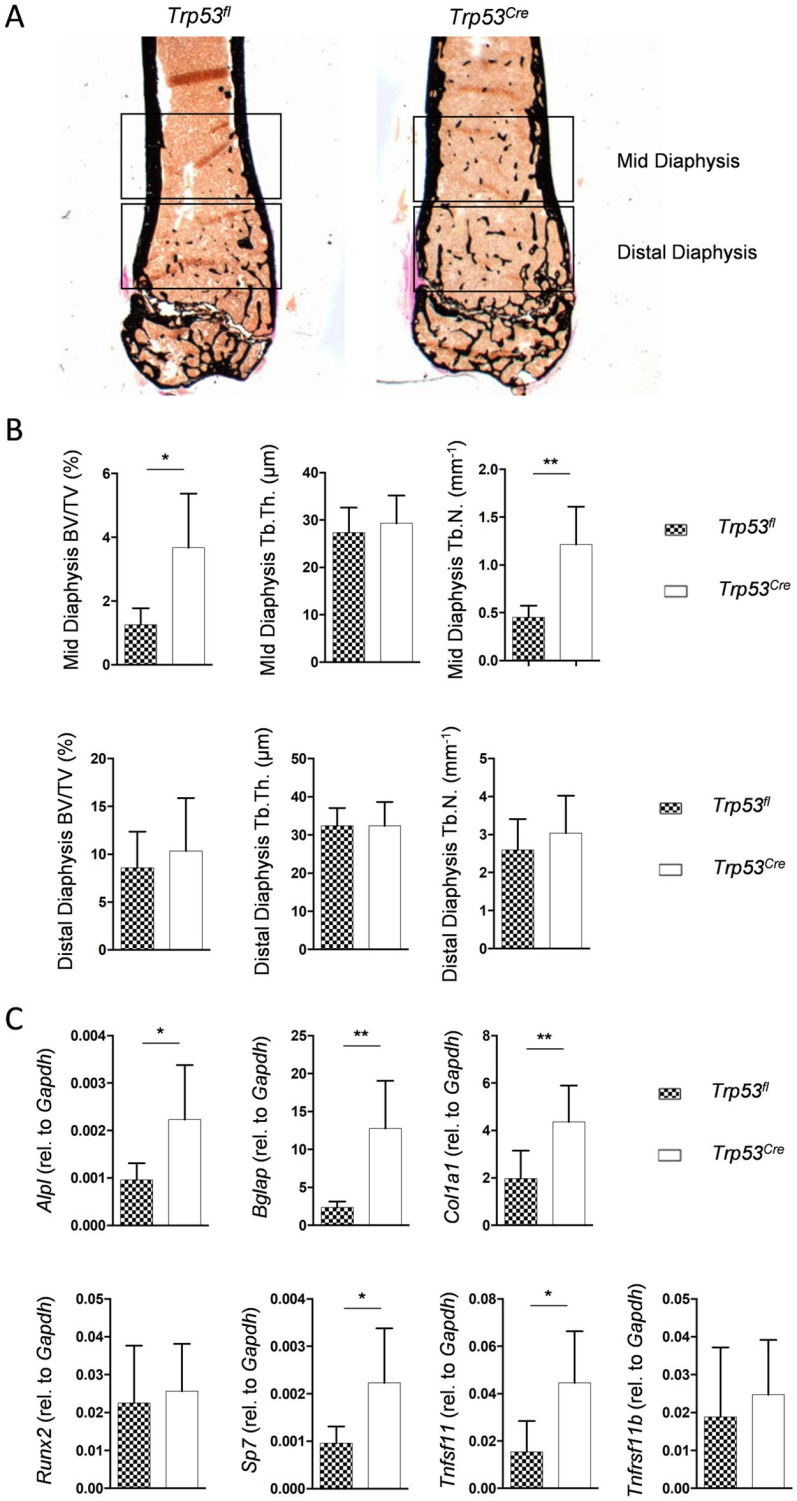
The *Trp53*^*Cre*^ phenotype is confirmed by femur histology and gene expression analysis. (A) Representative images of undecalcified femur sections from 6-month-old male mice with the indicated genotypes. Mineralized bone appears in black. The highlighted regions were separately analyzed. (B) Quantification of BV/TV, trabecular thickness (Tb.Th.) and trabecular number (Tb.N.) in the mid diaphysis (top) or the distal diaphysis (bottom) in 6-month-old male mice with the indicated genotypes. (C) qRT-PCR analysis for expression of the indicated genes in the femur, relative to *Gapdh*. Data represent mean ± SD (n ≥ 6). Asterisks indicate statistically significant differences (*p<0.05, **p<0.005).

At that point we decided to focus on the *Trp53*^*Cre*^ model in order to support the μCT findings. Here we first analyzed undecalcified femur sections from *Trp53*^*fl*^ and *Trp53*^*Cre*^ littermate mice (Fig 3A). We identified increased trabecular bone mass in the mid diaphysis of *Trp53*^*Cre*^ mice, but no significant difference towards controls in the distal diaphysis (Fig 3B). To substantiate these findings at the level of gene expression we dissected the femora and removed the epiphysis as well as the bone marrow by centrifugation. We then prepared RNA from the femoral bones for qRT-PCR expression analysis. As expected, we observed increased expression of osteoblast markers (*Alpl*, *Bglap* and *Col1a1*) in femora of *Trp53*^*Cre*^ mice (Fig 3C). Consistent with a previous report on *Trp53*-deficient mice and experiments with primary calvarial osteoblasts [20], we also observed that *Sp7* (encoding the transcription factor Osterix) was expressed at higher levels in *Trp53*^*Cre*^ mice. We additionally identified significantly higher expression of *Tnfsf11* (encoding RANKL), whereas expression of *Tnfrsf11b* (encoding osteoprotegerin) was not different between *Trp53*^*fl*^ and *Trp53*^*Cre*^ mice, unlike it was reported by others for p53-deficient mice [21]. We further used the bone marrow for FACS analysis, where we analyzed the CD45-positive cell populations. Despite a moderate increase of CD11c/CD45^+^ dendritic cells however, we did not observe significant differences for the most abundant cell types (S1 Fig).

**Fig 4:**
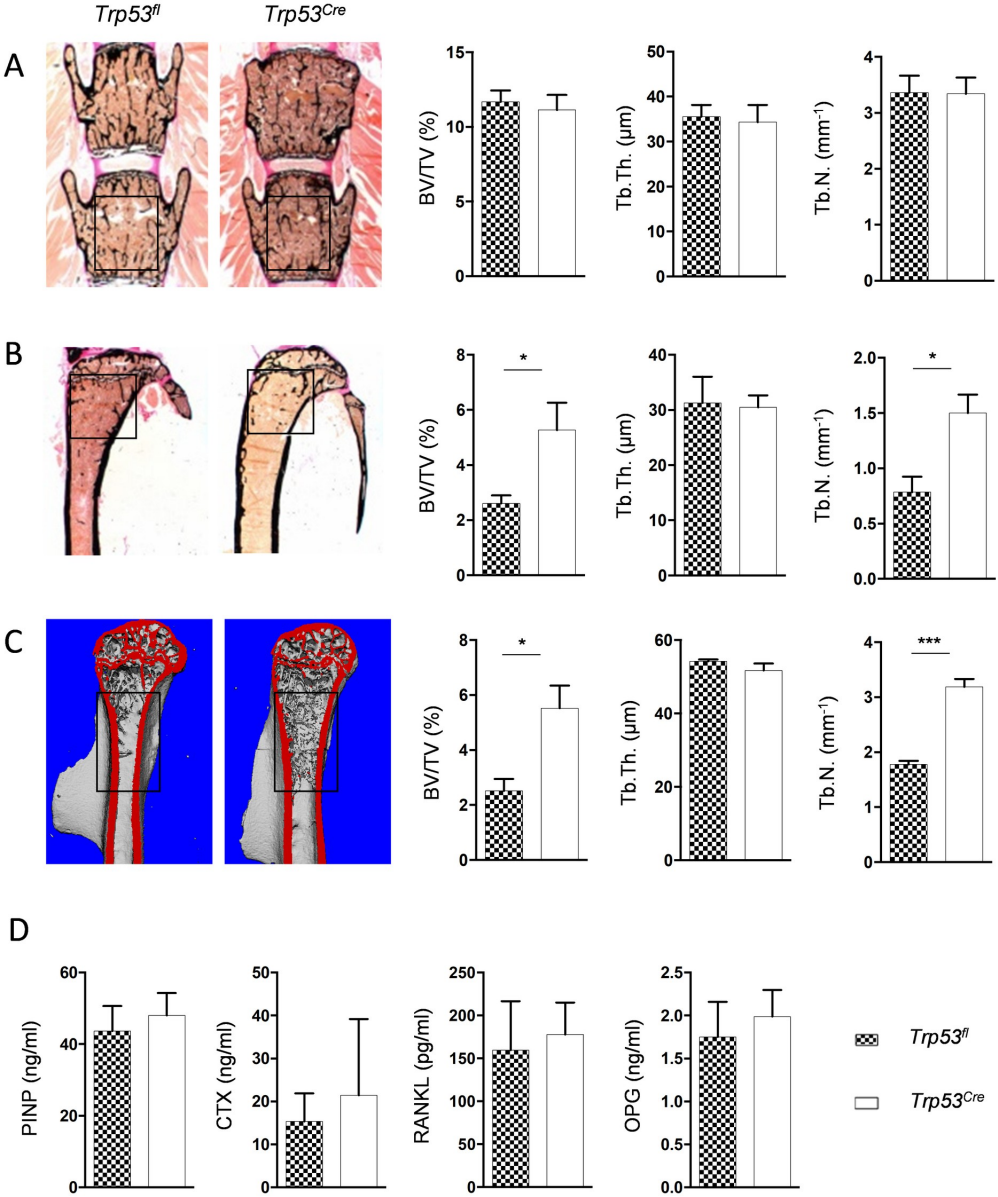
The *Trp53*^*Cre*^ bone phenotype is site-specific. (A) Representative images of undecalcified spine sections from from 6-month-old male mice with the indicated genotypes. Quantification of BV/TV, Tb.Th. and Tb.N., measured in the indicated region, is shown on the right. (B) Representative images of undecalcified tibia sections from the same mice. Quantification of BV/TV, Tb.Th. and Tb.N., measured in the indicated region, is shown on the right. (C) Representative μCT images of humeri from the same mice. Quantification of BV/TV, Tb.Th. and Tb.N., measured in the indicated region, in shown on the right. (D) Serum concentrations of PINP, CTX, RANKL and OPG in the same groups of mice. Data represent mean ± SD (n ≥ 6). Asterisks indicate statistically significant differences (*p<0.05, ***p<0.0005).

To address the question, if the increased trabecular bone mass is only observed in femora or also in other skeletal elements of *Trp53*^*Cre*^ mice, we additionally analyzed spine and tibia sections. Whereas no differences in trabecular bone parameters were observed between *Trp53*^*fl*^ and *Trp53*^*Cre*^ littermate mice for the spine (Fig 4A), there was significantly increased trabecular bone mass in the tibia of *Trp53*^*Cre*^ animals, albeit this phenotype did not extend into the mid diaphysis (Fig 4B). Therefore, we additionally performed μCT scanning of the humerus, which can be regarded as the forearm equivalent of the femur. Here we again identified an increased amount of trabecular bone in the mid diaphysis of *Trp53*^*Cre*^ mice (Fig 4C). Finally, we measured serum levels of the bone turnover biomarkers PINP and CTX, as well as RANKL and OPG concentrations, but here we did not detect significant differences between *Trp53*^*fl*^ and *Trp53*^*Cre*^ mice (Fig 4D). Taken together, these findings demonstrated that *Trp53*^*Cre*^ mice do not display a systemic high bone mass phenotype and suggested that p53 is physiologically required to limit trabecular bone formation in specific anatomical locations.

### *Trp53*^*Cre*^ bone marrow cells display increased osteogenesis, colony formation and proliferation

**Fig 5:**
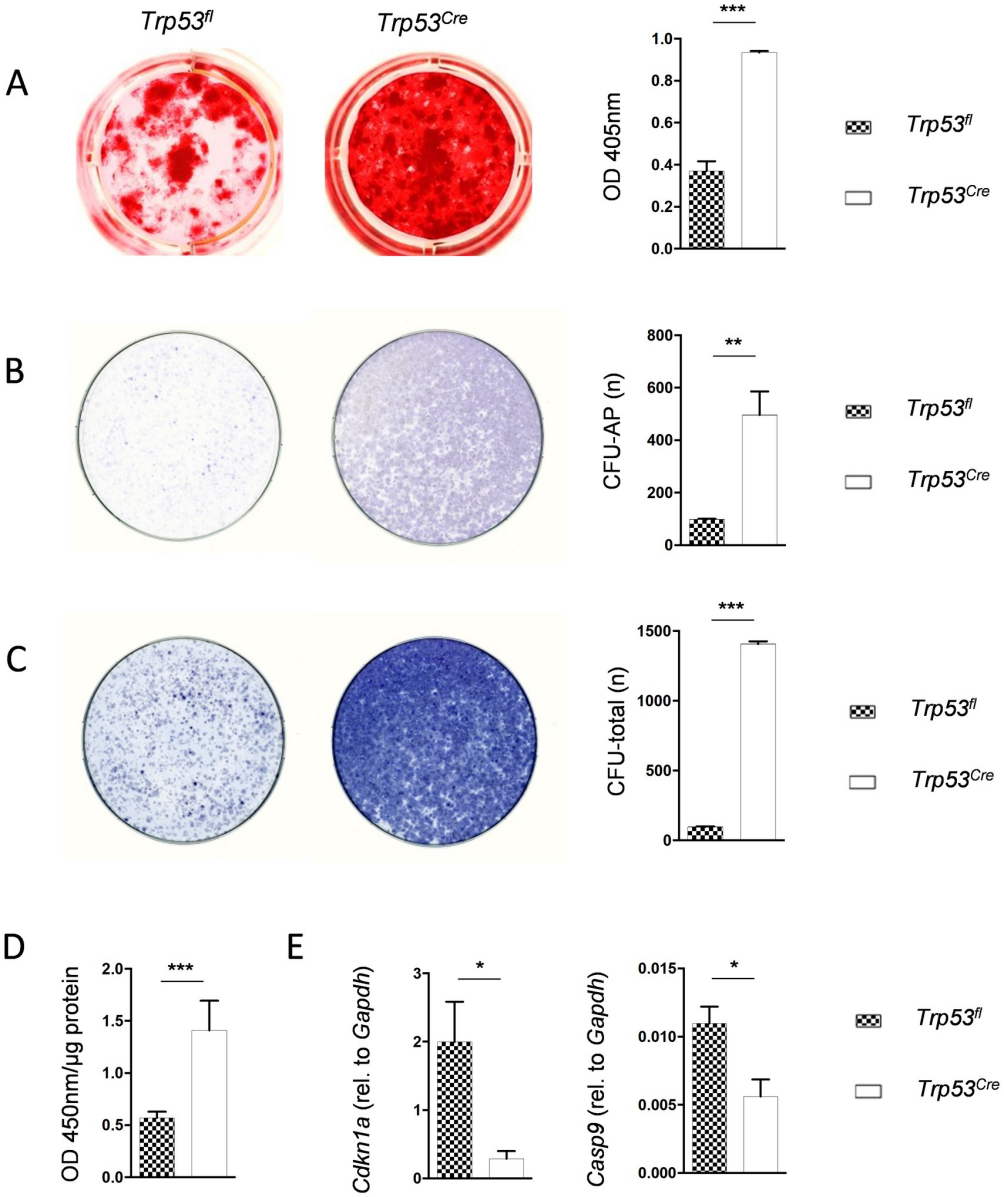
*Trp53*^*Cre*^ bone marrow cells display increased osteogenesis, colony formation and proliferation. (A) Representative images of cultured bone marrow cells from mice of the indicated genotypes, where mineralized matrix was stained by alizarin red at day 10 of osteogenic differentiation. Photometric quantification is shown on the right. (B) AP (alkaline phosphatase) activity staining of osteogenic colonies in cultured bone marrow cells from mice of the indicated genotypes two weeks after plating at low density. Quantification of the colony numbers is shown on the right. (C) Hematoxylin staining of all colonies in the same cultures. Quantification of the colony numbers is shown on the right. (D) BrdU incorporation assay in undifferentiated bone marrow cells from mice of the indicated genotypes. (E) qRT-PCR analysis for *p21* and *Casp9* expression, relative to *Gapdh*, in undifferentiated bone marrow cells from mice of the indicated genotypes. Data represent mean ± SD (n ≥ 6). Asterisks indicate statistically significant differences (*p<0.05, **p<0.005, ***p<0.0005).

We next isolated bone marrow cells from long bones of *Trp53*^*fl*^ and *Trp53*^*Cre*^ mice and studied their behavior *ex vivo*. Here we first assessed their osteogenic differentiation capacity by alizarin red staining of mineralized matrix formed after 10 day of culture in the presence of ascorbic acid and ß-glycerophosphate (Fig 5A). We hereby identified more than two-fold increase of mineralization in *Trp53*^*Cre*^ cultures. We additionally plated the bone marrow cells at lower density to let them establish colonies. By staining the osteogenic colonies for alkaline phosphatase we again observed a remarkable increase in *Trp53*^*Cre*^ cultures (Fig 5B). Importantly however, the increased colony forming capacity of *Trp53*^*Cre*^ bone marrow cells was not restricted to osteogenic colonies, since the total colony number was also substantially increased (Fig 5C).

This finding led us to analyze the proliferation of bone marrow cells immediately after plating. Here we identified, using BrdU incorporation assay, a more than 2-fold increased proliferation rate in *Trp53*^*Cre*^ cultures (Fig 5D). Consistently, expression of the p53 target gene *Cdkn1a*, encoding the cell cycle inhibitor p21 [22], was strongly repressed in *Trp53*^*Cre*^ cultures, and the same was the case for Casp9 [23], which is required for p53-dependent apoptosis (Fig 5E). We additionally performed the same set of experiments with bone marrow cells from Rsk2-deficient *Trp53*^*fl*^ and *Trp53*^*Cre*^ mice. Here we observed the same differences of osteogenesis, colony formation, proliferation and gene expression between bone marrow cultures from *Trp53*^*Cre;Rsk2-/0*^ and *Trp53*^*fl;Rsk2-/0*^ mice (S2 Fig). Taken together, these findings provided further evidence for a role of p53 as a negative regulator of osteoblastogenesis. They additionally suggested that the correction of the *Trp53*^*Cre*^ high bone mass phenotype by Rsk2 deficiency is not explained by the lack of a direct molecular interaction between p53 and Rsk2.

### The dental phenotype of *Rsk2*-deficient mice is not corrected by *Runx2-Cre*-mediated p53 inactivation

**Fig 6:**
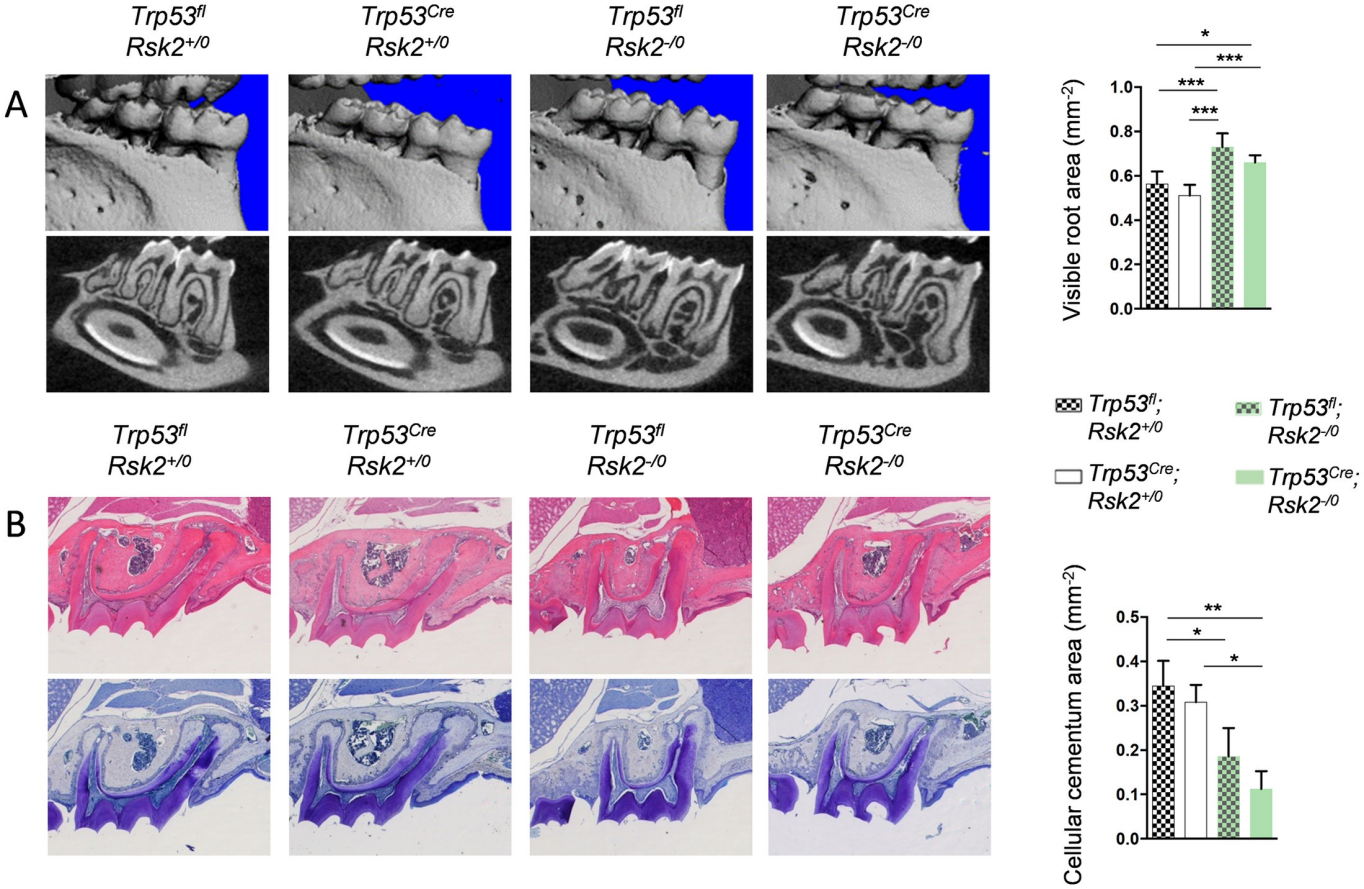
The dental phenotype of *Rsk2*-deficient mice is not corrected by *Runx2-Cre* -mediated p53 inactivation. (A) Representative μCT images of mandibular molars (top) and corresponding grayscale images (bottom) from 6-month-old male mice with the indicated genotypes. Quantification of the visible root area is shown on the right. (B) Hematoxylin (top) and toluidine blue (bottom) staining of molars form the same mice. Quantification of the cellular cementum area is shown on the right. Data represent mean ± SD (n ≥ 6). Asterisks indicate statistically significant differences (*p<0.05, **p<0.005, ***p<0.0005).

Since it was reported that Rsk2 phosphorylates p53 at Ser15, thereby acting upstream of p53 to promote a DNA damage response [24], it was not expected that the lack of such phosphorylation would affect the phenotype caused by p53 deficiency. On the other hand, it would have been possible that an alteration of p53 activity in *Rsk2*-deficient mice would contribute to their phenotypes. Since *Trp53*^*Cre*^ mice displayed a Rsk2-independent trabecular bone phenotype, we therefore focused on another pathologically affected tissue in *Rsk2*-deficient mice, i.e. the dental apparatus [12]. By assessing the dental phenotypes in the four groups of mice (*Trp53*^*fl;Rsk2+/0*^; *Trp53*^*Cre;Rsk2+/0*^; *Trp53*^*fl*;*Rsk2-/0*^; *Trp53*^*Cre;Rsk2-/0*^) by μCT imaging we identified alveolar bone loss, indicated by the visible root area, in *Rsk2*-deficient animals, regardless of their *Trp53* status (Fig 6A). Similarly, both *Rsk2*-deficient groups displayed a reduced area of cellular cementum (Fig 6B), which is a specialized hard tissue maintaining the teeth in the alveolar bone. These data indicate that the dental pathologies of *Rsk2*-deficient mice do not dependent on the presence of the Rsk2 substrate p53.

## Discussion

Oncogenic transformation of mesenchymal cells can cause osteosarcoma, the most prevalent primary bone tumor primarily affecting children or adolescents [25]. Osteosarcoma development is also a rare event in mice, but there is one particular mouse model displaying high incidence of osteosarcomas in different skeletal locations with 100% penetrance. More specifically, these mice carry a transgene causing ubiquitous over-expression of the oncogene *c-Fos* [13]. We have previously found that deficiency of the ribosomal S6 kinase 2 (Rsk2) strongly reduces osteosarcoma growth in cFos-transgenic mice [11]. However, since Rsk2 is known to phosphorylate two serine residues (Ser 362 and 374) of c-Fos, thereby stabilizing the protein to allow subsequent phosphorylation events, this remarkable influence of Rsk2 deficiency was to some extent expected. Therefore, it was important to address the question, if Rsk2 deficiency would also prevent osteosarcoma development in other mouse models.

For that purpose, we decided to take advantage of *Trp53*^*fl/fl*^ mice that were crossed with *Runx2-Cre*-transgenic animals to achieve p53 inactivation in osteoblast progenitor cells. We then generated four groups of mice (*Trp53*^*fl;Rsk2+/0*^; *Trp53*^*Cre;Rsk2+/0*^; *Trp53*^*fl*;*Rsk2-/0*^; *Trp53*^*Cre;Rsk2-/0*^) with the aim to analyze osteosarcoma progression. Unexpectedly, due to *Trp53* recombination in the thymus, we identified a high incidence of thymic lymphomas in *Trp53*^*Cre*^ mice, which did not allow us to let them get older in order to develop osteosarcomas. Importantly, however, when we analyzed the different groups of mice we identified another fully unexpected pathology in *Trp53*^*Cre*^ mice, i.e. increased trabecular bone formation in the midshaft of femur and humerus. Although such a phenotype is in principal agreement with previous evidence showing that p53 deficiency in mice enhances proliferation and osteogenic differentiation of mesenchymal stem cells to cause an increased bone mass phenotype [20, 21, 26–30], we are not aware of a conditional p53 inactivation model with such a specific localized influence on trabecular bone formation.

Skeletal development, growth and remodeling are highly complex processes, which are regulated by a variety of cell types and specific molecules [31]. Moreover, even after development and growth are completed, there is a steady remodeling of the bone matrix, which is a prerequisite for life-long skeletal stability [32]. This process is mediated by two antagonistically acting cell types, i.e. bone-resorbing osteoclasts and bone-forming osteoblasts [33, 34]. Importantly, these two cell types are fundamentally different, not only in terms of progenitor cells, morphology and mode of action but also with respect to specific molecules regulating their differentiation and function [35]. It is also noteworthy that there are two distinct compartments in adult bones, i.e. cortical and trabecular bone, the latter being remodeled at higher rate [36]. This architecture is of key importance, as it provides non-mineralized space for the bone marrow, which does not only contain hematopoietic cells, but also mesenchymal stromal cells, the latter representing a pool of osteoblast progenitor cells [37]. Especially in the long bones of the extremities, such as the femora or the humeri, there is essentially no trabecular bone in the midshaft region of the marrow cavity under physiological circumstances. Therefore, it is certainly possible to state that the phenotype observed in *Trp53*^*Cre*^ mice is an example of non-physiological or may be even ectopic bone formation. This implies that, at least in mice, p53 expression in osteoprogenitor cells is required to limit their bone-forming capacity at specific skeletal locations.

Another surprising observation was that additional Rsk2 deficiency apparently prevented the increased trabecular bone phenotype of *Trp53*^*Cre*^ mice. However, since *Rsk2*-deficient mice display a low bone mass phenotype, this finding did not specifically demonstrate a genetic interaction between p53 and Rsk2 in controlling bone formation. Therefore, it was important to compare the osteogenic potential of bone marrow cells derived from the four groups of mice. The results we obtained here were truly informative, since there was a remarkable difference between *Trp53*^*fl*^ and *Trp53*^*Cre*^ cultures, but no normalization of this difference by additional Rsk2 deficiency. Vice versa, we did not observe a correction of the dental phenotype in *Rsk2*-deficient mice by conditional inactivation of p53. From these latter findings it is certainly possible to conclude that the dental abnormalities caused by Rsk2 deficiency are not explained by the lack of Rsk2-mediated p53 phosphorylation.

Despite the serendipity of our findings, we recognize the limitations of the present study. First, as already stated above, we mainly confirm but also extent previous findings reported for p53-deficient mice or their respective osteoblast cultures. Second, we only used one *Cre*-expressing mouse line, which does not allow conclusions about the stage of osteoblast differentiation that is most critical for the function of p53. Third, we only analyzed unsorted bone marrow cells in *ex vivo* experiments, which did not allow to address the question, if p53 inactivation in lymphoid cells is a major driver of the increased osteogenesis we observed. Therefore, our findings provide the basis for future experimental approaches that should not only include the use of additional Cre-deleters to inactivate p53 at different stages of osteoblast differentiation, but also co-cultures of sorted bone marrow cell populations from *Trp53*^*fl*^ and *Trp53*^*Cre*^ mice. Especially since there is hallmark evidence for a crosstalk between bone and immune cells [38], such future experiments are certainly required to fully understand the complexities of p53 action in the bone marrow, but exceeds the scope of this manuscript.

Regardless of these limitations, and despite the fact that our findings did not allow us to study the impact of Rsk2 deficiency on osteosarcoma development, it is important that we observed a fully unexpected and highly specific skeletal phenotype in *Trp53*^*Cre*^ mice. Our collective data demonstrate that p53, in a Rsk2-independent manner, is required to limit trabecular bone accumulation in the appendicular skeleton, at least in mice.

## Supporting information

S1 Fig Comparative analysis of hematopoietic lineages in bone marrows of *Trp53*^*Cre*^ *and Trp53*^*fl*^ mice. FACS analysis of CD45-positive leukocytes in bone marrow of *Trp53*^*fl*^ and *Trp53*^*Cre*^ mice for specific surface markers, as indicated. The percentage is given for hematopoietic progenitor cells (C34/CD45^+^), monocytes/macrophages/granulocytes (CD11b/CD45^+^), macrophages (F4/80/CD45^+^), dendritic cells (CD11c/CD45^+^), B cells (C45R/CD45^+^), and T cells (C3e/CD45^+^ and C4/CD45^+^). Data represent mean ± SD (n = 3). The asterisk indicates a statistically significant difference (*p<0.05).
(TIF)

S2 Fig Increased osteogenesis, colony formation and proliferation of *Trp53*^*Cre*^ bone marrow cells is independent of Rsk2. (A) Representative images of cultured bone marrow cells from mice of the indicated genotypes, where mineralized matrix was stained by alizarin red at day 10 of osteogenic differentiation. Photometric quantification is shown on the right. (B) AP (alkaline phosphatase) activity staining of osteogenic colonies in cultured bone marrow cells from mice of the indicated genotypes two weeks after plating at low density. Quantification of the colony numbers is shown on the right. (C) Hematoxylin staining of all colonies in the same cultures. Quantification of the colony numbers is shown on the right. (D) BrdU incorporation assay in undifferentiated bone marrow cells from mice of the indicated genotypes. (E) qRT-PCR analysis for *p21* and *Casp9* expression, relative to *Gapdh*, in undifferentiated bone marrow cells from mice of the indicated genotypes. Data represent mean ± SD (n ≥ 6). Asterisks indicate statistically significant differences (*p<0.05, **p<0.005, ***p<0.0005).
(TIF)
