# Supplementary figures and images for: Osteoblast-specific inactivation of p53 results in locally increased bone formation
Source: PLoS One. 2021 Nov 18;16(11):e0249894. doi: 10.1371/journal.pone.0249894 (PMC8601510; doi:10.1371/journal.pone.0249894)

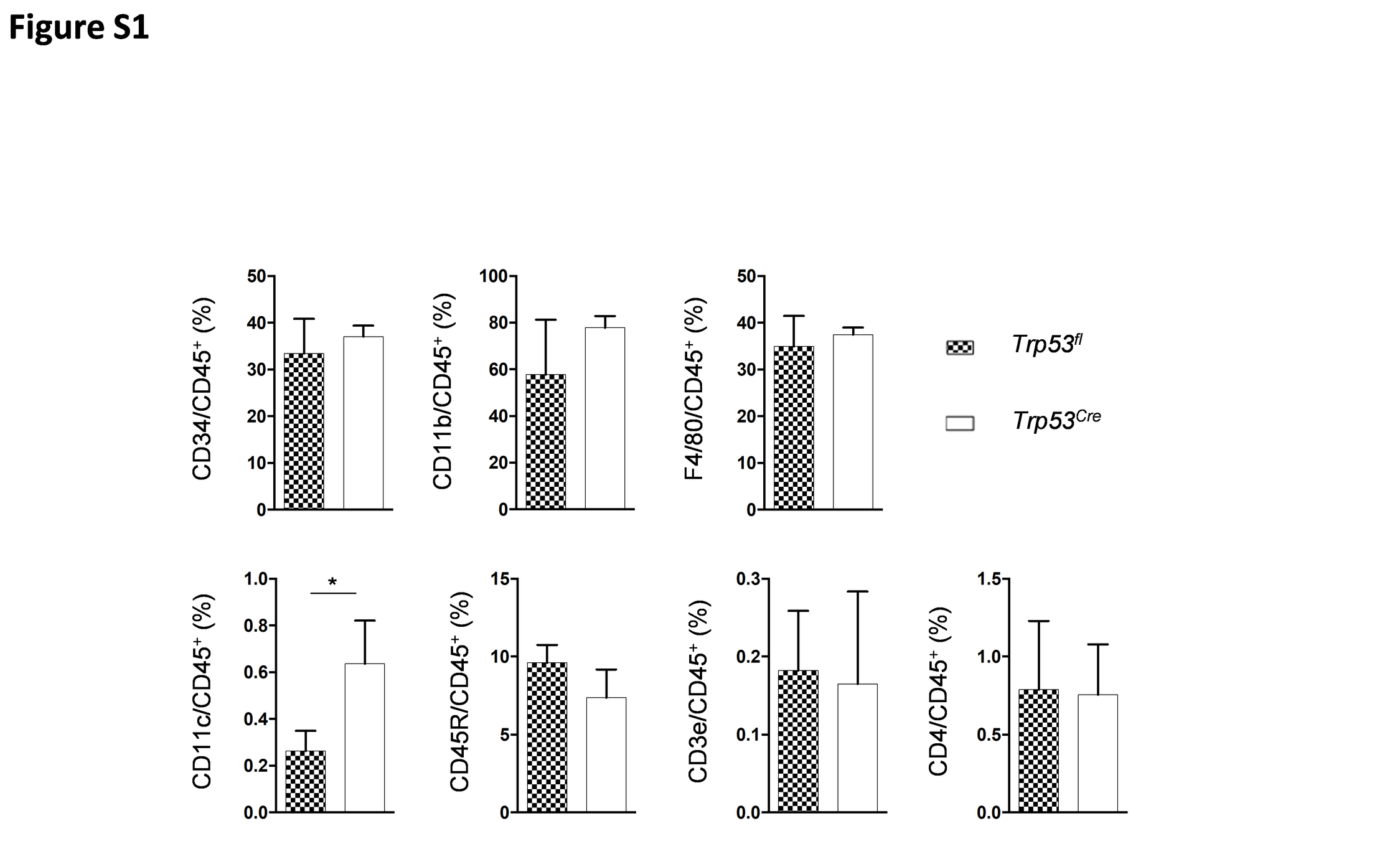

Supplement: S1 Fig — FACS analysis of CD45-positive leukocytes in bone marrow of Trp53fl and Trp53Cre mice for specific surface markers, as indicated. The percentage is given for hematopoietic progenitor cells (C34/CD45+), monocytes/macrophages/granulocytes (CD11b/CD45+), macrophages (F4/80/CD45+), dendritic cells (CD11c/CD45+), B cells (C45R/CD45+), and T cells (C3e/CD45+ and C4/CD45+). Data represent mean ± SD (n = 3). The asterisk indicates a statistically significant difference (*p<0.05). (TIF) [file pone.0249894.s001.tif]

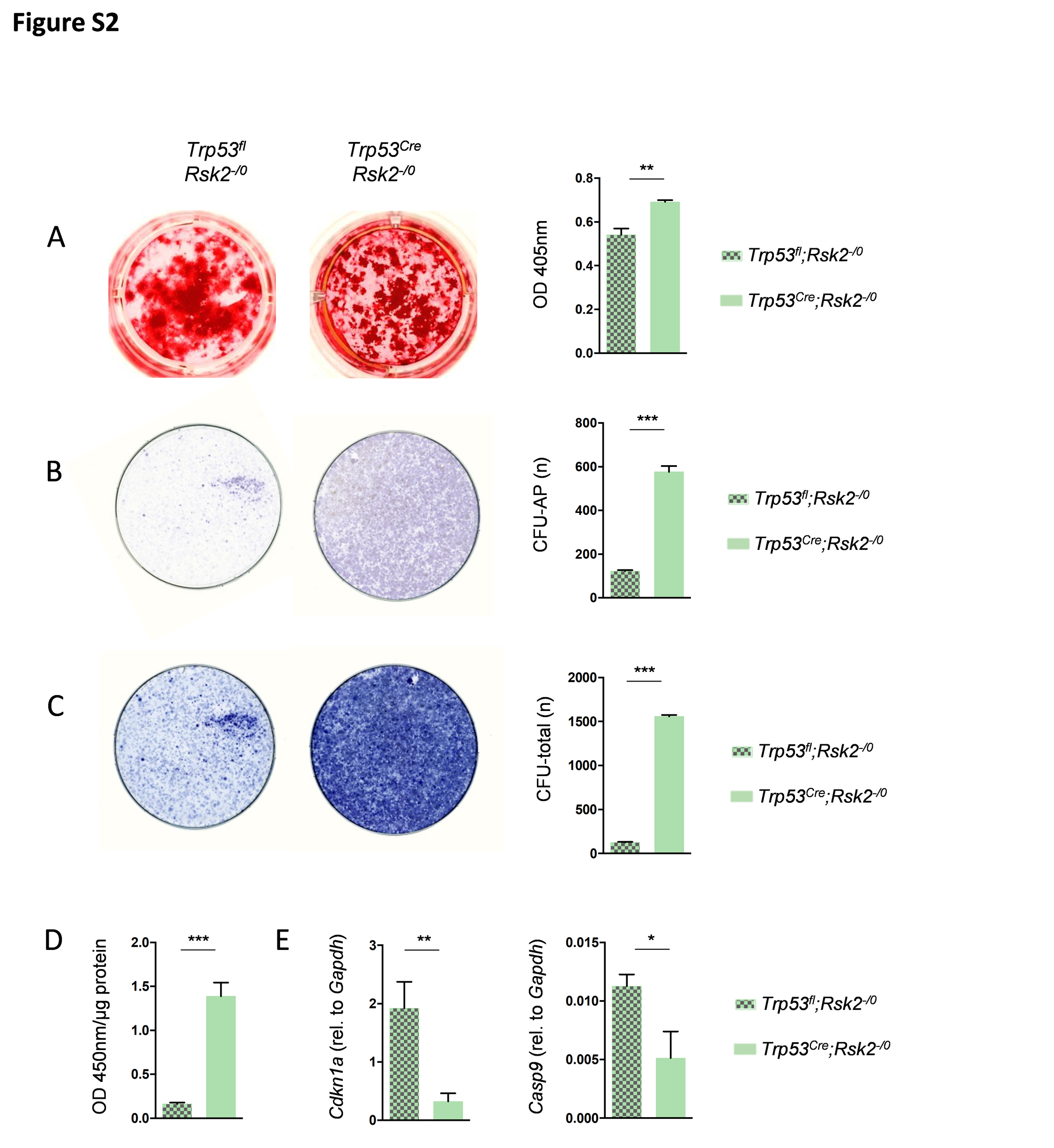

Supplement: S2 Fig — (A) Representative images of cultured bone marrow cells from mice of the indicated genotypes, where mineralized matrix was stained by alizarin red at day 10 of osteogenic differentiation. Photometric quantification is shown on the right. (B) AP (alkaline phosphatase) activity staining of osteogenic colonies in cultured bone marrow cells from mice of the indicated genotypes two weeks after plating at low density. Quantification of the colony numbers is shown on the right. (C) Hematoxylin staining of all colonies in the same cultures. Quantification of the colony numbers is shown on the right. (D) BrdU incorporation assay in undifferentiated bone marrow cells from mice of the indicated genotypes. (E) qRT-PCR analysis for p21 and Casp9 expression, relative to Gapdh, in undifferentiated bone marrow cells from mice of the indicated genotypes. Data represent mean ± SD (n ≥ 6). Asterisks indicate statistically significant differences (*p<0.05, **p<0.005, ***p<0.0005). (TIF) [file pone.0249894.s002.tif]
